# Household Transitions to Clean Energy in a Multi-Provincial Cohort Study in China

**DOI:** 10.1038/s41893-019-0432-x

**Published:** 2019-11-25

**Authors:** Ellison Carter, Li Yan, Yu Fu, Brian Robinson, Frank Kelly, Paul Elliott, Yangfeng Wu, Liancheng Zhao, Majid Ezzati, Xudong Yang, Queenie Chan, Jill Baumgartner

**Affiliations:** a Department of Civil and Environmental Engineering, Colorado State University, 1372 Campus Delivery, Fort Collins, CO, USA 80524; b Institute on the Environment, University of Minnesota, 1954 Buford Avenue, Saint Paul, MN, USA, 55108; c Department of Epidemiology and Biostatistics, School of Public Health, Imperial College London, Norfolk Place St. Mary’s Campus, London, UK W2 1PG; d Department of Analytical, Environmental & Forensic Sciences, School of Population Health & Environmental Sciences, Kings College London, 150 Stamford Street, London, UK SE1 1UL; e Department of Building Science, School of Architecture, Tsinghua University, 1 QingHua Yuan Road, Beijing, China 100084; f Department of Geography, McGill University, 805 Sherbrooke Street West, Montreal, Canada H3A 0B9; g MRC-PHE Centre for Environment and Health, School of Public Health, Imperial College London, Norfolk Place St. Mary’s Campus, London, UK W2 1PG; h Peking University Clinical Research Institute, 38 Xueyuan Road, Beijing, China 100191; i National Center for Cardiovascular Disease, Fuwai Hospital, Peking Union Medical College & Chinese Academy of Medical Sciences, Beijing, China 100006; j Department of Epidemiology, Biostatistics, and Occupational Health, McGill University, 1110 Pine Avenue West, Montreal, Canada H3A 1A3

**Keywords:** solid fuel suspension, household air pollution, electrification, stove adoption

## Abstract

Household solid fuel (biomass, coal) burning contributes to climate change and is a leading health risk factor. How and why households stop using solid fuel stoves after adopting clean fuels has not been studied. We assessed trends in the uptake, use, and suspension of household stoves and fuels in a multi-provincial cohort study of 753 Chinese adults and evaluated determinants of clean fuel uptake and solid fuel suspension. Over one-third (35%) and one-fifth (17%) of participants suspended use of solid fuel for cooking and heating, respectively, during the past 20 years. Determinants of solid fuel suspension (younger age, widowed) and of earlier suspension (younger age, higher education, and poor self-reported health status) differed from the determinants of clean fuel uptake (younger age, higher income, smaller households, and retired) and of earlier adoption (higher income). Clean fuel adoption and solid fuel suspension warrant joint consideration as indicators of household energy transition. Household energy research and planning efforts that more closely examine solid fuel suspension may accelerate household energy transitions that benefit climate and human health.

China has made major investments in air pollution reduction over the past decade (1). As air pollution from industry and traffic decrease, China’s ability to meet domestic and global air pollution standards requires a shift in household energy from high-polluting solid fuel (i.e., coal, biomass) stoves to clean fuels like gas and electricity (2). Suspension of solid fuel use is essential to reducing environment-related disease burden in China and other low- and middle-income countries and to achieving global Sustainable Development Goals (SDGs; e.g., proportion of the population primarily using clean fuels (7.1.2); levels of urban air pollution (11.6.2); and mortality associated with household and outdoor air pollution (3.9.1)) (3, 4).

Solid fuel stoves are used by over 2.5 billion people for cooking, heating, lighting, and other energy needs (5). Emissions from these stoves contribute to air pollution levels that are 2-8 times higher than the WHO air quality interim target (level 1) for fine particles (6). Air pollution from solid fuel stoves contributes to an estimated 2.8 million premature annual deaths (7) and influences regional and global air quality and climate (8, 9). Health studies indicate non-linear associations between air pollution and key health outcomes, including cardiovascular diseases and childhood pneumonia, meaning that the greatest health benefits accrue only when achieving low exposure levels below the WHO guideline (10). Partial reductions in solid fuel stove use are unlikely to lower air pollution levels to those that greatly minimize adverse health impacts (11).

Gas fuels and electricity, at their point of use, are the lowest polluting forms of household energy (12). Liquefied petroleum gas (LPG) and other gases (e.g., ethanol, biogas) are increasingly available and used in most low-income settings (13), and an estimated 78% of rural homes globally have access to electricity (14). However, complete transition to clean fuels requires more than a stove technology upgrade, but also a shift in user behavior, adaptation of cultural preferences, and, ultimately, giving up solid fuel stoves that may have many perceived benefits (15). Households that adopt clean stoves often concurrently use their solid fuel stoves for years and even decades (16, 17), a practice driven by fuel prices and the perceived suitability of stoves for different tasks (18–20). Combined use of traditional and clean stoves limits the air pollution reductions that are achievable (11), and can even lead to higher air pollution (21–24), underscoring the importance of exclusive or near-exclusive use of clean fuels.

Patterns and drivers of, as well as barriers to, adoption and sustained use of clean stoves are increasingly well documented in field studies worldwide (16, 25–28). Systematic reviews of the literature have evaluated the influences of household (e.g. demographics, socioeconomic status, occupation), local environmental (geography, climate), and external conditions (e.g. policies, fuel and stove prices, financial incentives, fuel supply) in clean stove and fuel adoption (29, 30), which can vary in magnitude and direction across settings (31). In contrast, suspension of solid fuels, an essential complement to clean fuel adoption in the energy transition process, remains virtually unstudied, as has the timing of major household-level energy choices in settings with persistent solid fuel use. Investigating when, not just whether, a household makes a change in their household energy composition may reveal household- and village-level factors that could be leveraged better to accelerate household energy transitions. A better understanding of the solid fuel suspension process, and what factors may hasten or hinder it, would support policy and planning efforts that accelerate the more complete clean energy transitions required to achieve the SDGs.

Leveraging data from the INTERMAP China Prospective (ICP) study, a longitudinal study of environmental risk factors for disease in 3 geographically diverse provinces, we conducted the first study to investigate the long-term patterns and determinants of clean fuel uptake and solid fuel suspension among rural and peri-urban Chinese households. Our study provides new insights into the household energy transition process that can inform the planning and implementation of large-scale rural energy programs aimed at reducing the environmental and disease burden of household solid fuel burning.

## Results

A total of 753 (96%) of participants enrolled into the present study in 2016 (n=784) completed household energy surveys, including 246, 284, and 223 participants in Beijing, Shanxi, and Guangxi, respectively. The 31 participants without complete surveys (13 in Beijing, 6 in Shanxi, and 12 in Guangxi) were mostly (94%) original enrollees in the INTERMAP study with limited mobility and were thus re-enrolled in their homes rather than at the central location where the energy questionnaires were conducted. Site-specific socio-demographic information is summarized in Supplementary Table 1. Reporting on heating fuel in this study focuses on Beijing and Shanxi where cold winters necessitate space heating.

### Household energy use patterns

All participants cooked with solid fuel stoves at baseline (i.e., 20 years ago, in 1995-1997), and the majority (72%) of those living in northern provinces (Beijing, Shanxi) were also heating with solid fuel (the other 28% did not report using heating stoves at baseline). Since then, over a third (35%) of participants reported suspending use of solid fuel for cooking, which was double the proportion of those who suspended use of solid fuel for heating (17%) (Table 1). None of the participants in Guangxi, a subtropical region with mild winters, reported any use of solid fuel heating fuels or devices and only a few (n=14) acquired clean heating devices over the study period. Just 54 (10%) of participants living in northern China reported complete suspension of solid fuel for both cooking and heating. Including the 68 participants in Guangxi who transitioned to exclusive use of clean cooking fuel (and who did not use energy for home heating), 16% of the study population fully suspended use of solid fuel.

**Figure 1:**
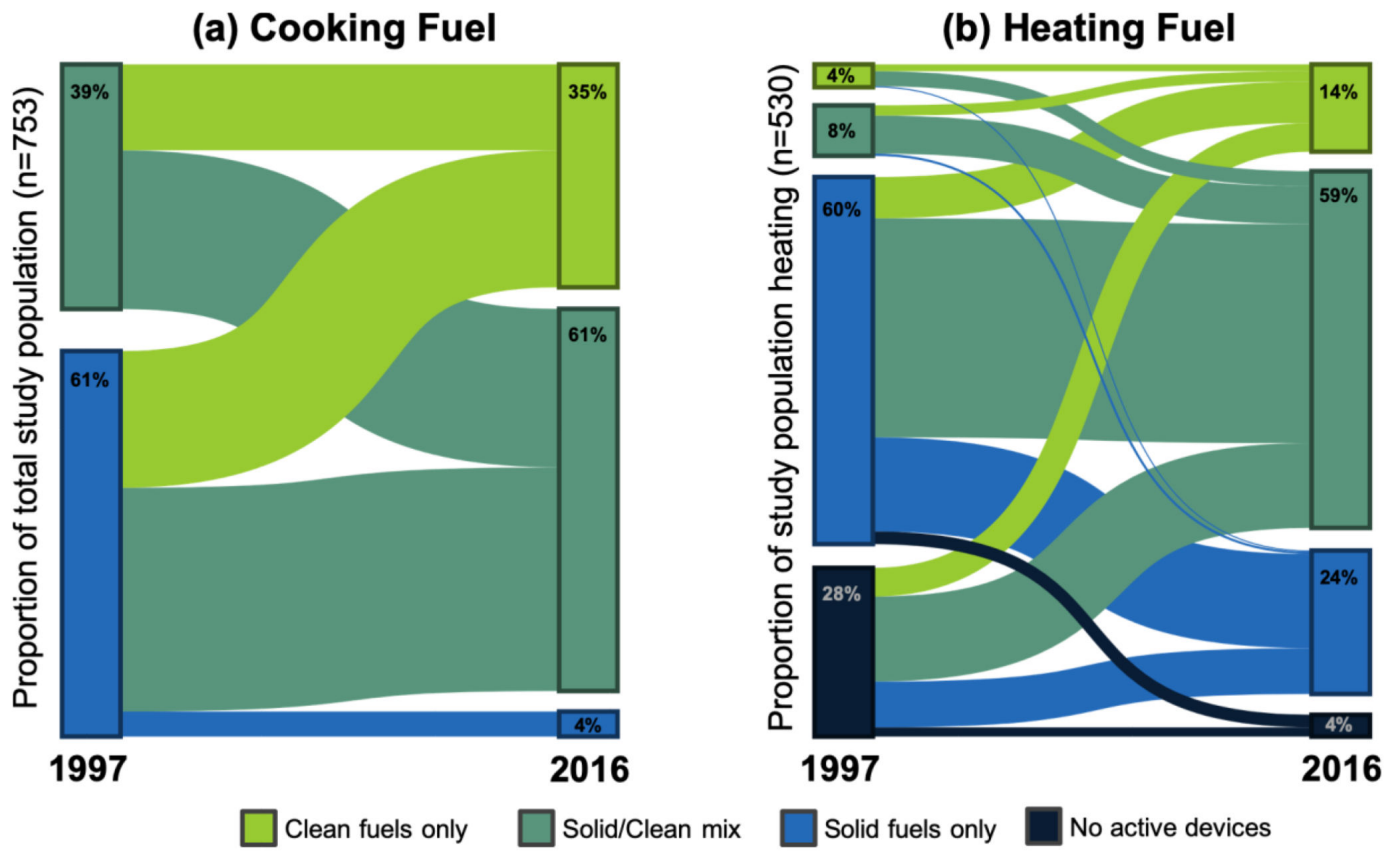
Proportion of the study population at baseline (1997) and at follow-up (2016) exclusively using solid fuel (blue), using both solid and clean fuel (teal), and exclusively using clean fuel (green) for cooking (a) and heating (b). The heating fuel transition also includes a category at baseline and at present for study participants who did not report use of any fuels or device for space heating (dark blue).

**Figure 2:**
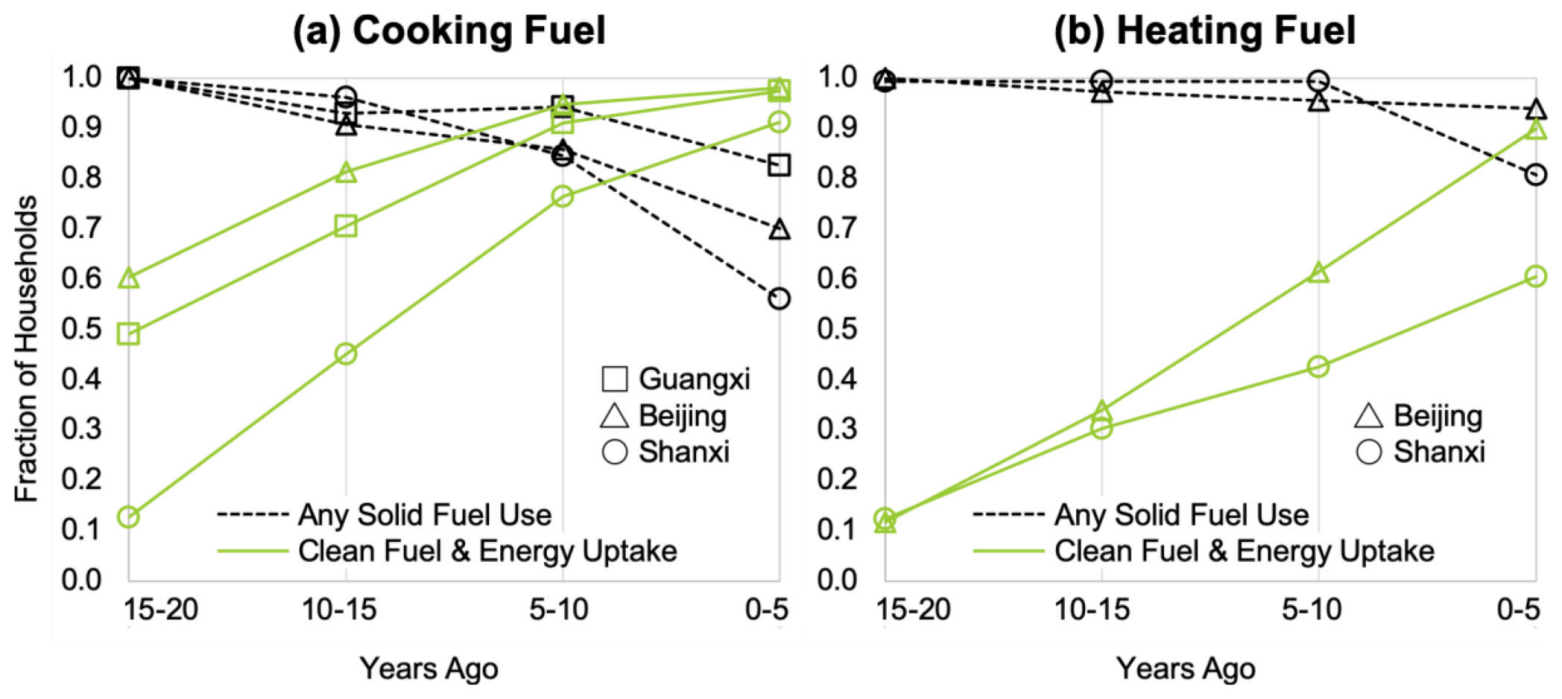
Temporal trends, evaluated at 5-year intervals, in any solid fuel use (dashed black lines) and clean fuel uptake (solid green lines) for cooking activities (a) in each of the 3 study sites and heating activities (b) in the 2 northern provinces (Beijing and Shanxi).

The household energy transition for this study population was dominated by the uptake of clean energy and a switch from exclusive use of solid fuel to combined use of both solid and clean fuels (i.e., mixed use) (Table 1) with evidence for nearly all possible baseline-to-followup pathways (Figure 1). The rate of clean fuel uptake was higher and more consistent than the rate of solid fuel suspension (Figure 2). This was especially evident for heating, where solid fuel suspension was modest (0% to 17% over the 20-year follow-up period) compared with changes in use of clean heating fuels over the same time period (10% to 73%). In Shanxi, the proportion of participants using clean cooking fuel rose from 13% at baseline to 91% in 2016. The proportion of households using clean cooking fuel was higher at baseline and in 2016 in Beijing (60%; 98%) and Guangxi (49%; 97%) compared with Shanxi. Yet, the proportion of households suspending use of solid cooking fuel in Shanxi was greater than in Beijing and Guangxi. Similarly, suspension of solid fuel for heating in Shanxi (20%) was more than double what it was in Beijing (9%), despite the prevalence of clean heating fuel use being considerably lower overall in Shanxi (59%) compared to Beijing (90%). Some participants living in Beijing and Shanxi, where wintertime temperatures warrant daily heating, were not using any fuels for heating 20 years ago. While it is possible they used solid fuels more than 20 years ago (i.e., before baseline) and ceased using those fuels for a period of time before taking up heating fuel use again, it is more likely that they were surviving without heating fuels, and later chose to start using heating fuels, including cleaner options.

**Figure 3:**
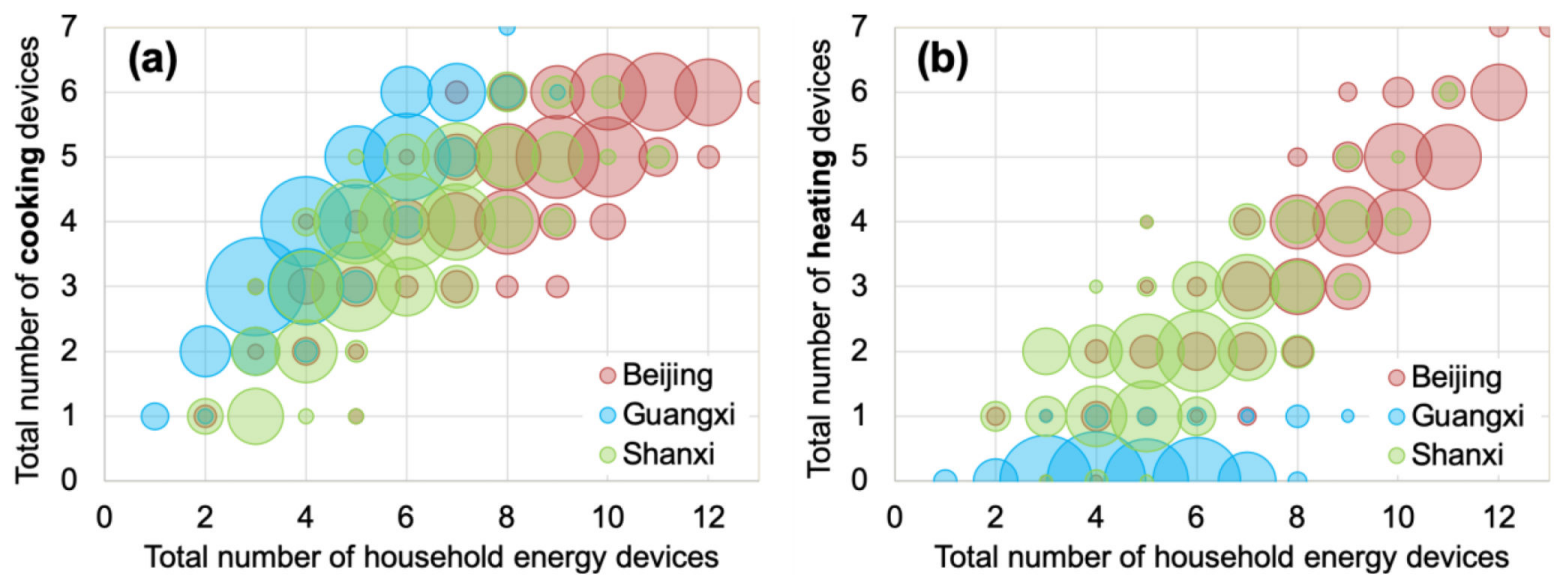
Total number of cooking (A) or heating (B) devices owned as a function of total household energy devices reported. Marker size is proportional to the number of participants.

Across all sites, participants used 15 different solid fuel devices (cooking n=7; heating n=8) and 14 different gas or electric devices (cooking n=8; heating n=4; water-boiling n=2) (Supplementary Figure 1). Within households, participants reported using 1 to 13 household energy devices (1-7 cooking devices, 0-7 heating devices). Beijing participants had, on average, more devices (mean ± standard deviation: 8.7 ± 2.2) than those in Shanxi (5.8 ± 1.8) or Guangxi (4.6 ± 1.5) (Figure 3), with participants in all provinces reporting more cooking than heating devices, on average (Supplementary Figure 2).

Our analysis separately analyzed *whether* households ever decide to adopt clean fuel or suspend solid fuel use and then, for households that do make that choice, *when* they decide to do this. These two decision-making processes seem to be driven by different factors.

### Determinants of clean fuel uptake and solidfuel suspension

The determinants of solid fuel suspension differed from clean fuel uptake, and also differed for cooking versus heating (Table 2). Being younger or widowed was associated with suspending use of solid fuel for cooking. Being younger was also associated with adoption of clean cooking fuels, but, in addition, so was being retired or the member of a smaller household. Higher income, excluding the highest income bracket, was associated with adoption of clean cooking fuel, whereas higher income was not associated with suspension of solid cooking fuel except for the highest bracket. Higher income was also not associated with either suspension of solid heating fuel or uptake of clean heating fuel, though participants with higher education and who were retired were more likely to adopt clean heating fuels.

### Determinants of the timing of suspension and adoption

Factors associated with earlier suspension of solid fuel differed from those associated with earlier clean fuel adoption (Table 3). For cooking, being younger or attaining higher levels of education was associated with earlier suspension of solid fuel, but these factors were not associated with when households started using clean energy. Income was associated with earlier clean fuel adoption but not with solid fuel suspension. For cooking, only being in the highest income bracket was associated with earlier clean fuel adoption. In contrast, being in all but the highest income bracket was associated with later adoption of clean heating fuel. Participants who suspended use of solid fuel for heating did so earlier if they also reported being in poorer health, which may suggest a choice to reduce their exposure to indoor smoke. There was some evidence that, after adjusting for age, being one of the newly enrolled participants was associated with later adoption of clean cooking fuels but earlier suspension of solid fuel for heating. Finally, being an early adopter of a clean fuel (i.e., more time since uptake of clean fuel), which was associated with suspending solid fuel use at all (Table 3), was also associated with earlier solid fuel suspension (Table 3).

We adjusted for village of residence (n=13 villages) in all statistical models. These village-level fixed effects appeared important for whether and when participants suspended use of solid cooking or heating fuels, whether participants started using clean heating fuel, and when participants started using clean cooking fuel. Village-level unobservables were not associated with uptake of clean cooking fuel, likely because it is now nearly universal in China. Rates of solid fuel suspension were less gradual and more varied among all villages compared to clean fuel uptake, with several villages experiencing most solid fuel suspension within a 5-year time interval, and mostly within the most recent past 5 years (Supplementary Figures 3 and 4).

Our results did not change after re-classifying households reporting ‘rare use of solid fuel’ (i.e., near exclusive use) as solid fuel users. When we repeated our analysis with outcomes for suspension or uptake of heating and cooking fuels combined, we observed that being younger, widowed, or in the highest income bracket was associated with complete suspension of solid fuel use (Supplementary Table 2). Being younger, more highly educated, or an earlier adopter of clean cooking fuel was associated with earlier suspension (Supplementary Table 3). These results are similar to our findings for cooking fuel suspension in the main analysis, reflecting the larger proportion of the study population choosing to suspend solid cooking fuels compared with heating. However, among participants who suspended use of any solid fuel, being in the two highest income brackets was associated with later solid fuel suspension, potentially reflecting a preference for increased energy intensity over suspension of solid fuels as income increases.

## Discussion

In this first evaluation of household solid fuel suspension, the rate and prevalence of suspension is slower and lower than clean fuel uptake. Exclusive, or near-exclusive, use of clean energy was rare although nearly all participants started using some clean fuel for cooking and many started using clean fuel for space heating since 1997. Suspension of solid fuel for cooking was more common than for heating. Further, the factors that were associated with solid fuel suspension and the timing of that decision differed from clean fuel adoption. Collectively, our results suggest that joint consideration of clean fuel adoption and solid fuel suspension may be helpful in shaping new, constructive directions for research and policy related to household energy transitions in China and other countries.

Clean fuel uptake in our study households increased steadily during a period of dramatic socioeconomic transition in China (2, 32, 33). The last several decades were marked by major economic reforms and rural development programs including efforts to improve rural energy and the infrastructure required to deliver that energy to homes (34). Some rural energy programs were met with variable or limited success (e.g., small-scale hydro, fuelwood forests, biogas). Others like electrification, rural coal mining enterprises (35), and the dissemination of hundreds of millions of wood-chimney stoves during the National Improved Stove Program (initiated in the 1980s) (36) were highly successful and likely contributed to observed trends in clean fuel uptake and solid fuel suspension in our study. These results are consistent with recent studies of household energy transition in China, including a prospective study of 0.5 million urban and rural households over 5 decades in 10 Chinese provinces (~50% and 25% increase in the fraction of homes reporting primary use of clean fuels for cooking and heating, respectively) (37) and a national survey of primary cooking and heating fuel choices (40% and 20% reported primarily using a clean fuel for cooking and heating, respectively) (38). These studies evaluated only primary fuel use, making it difficult to gauge the extent to which homes continued using solid fuel (i.e., energy stacking). In this study, the time to onset of suspension did not coincide with the timing of clean fuel uptake; rather, our data show that people adopt clean fuels and increase household energy use intensity (i.e., use both clean and less clean concurrently) for years before starting to give up less clean fuels.

Our results support a broader literature showing that concurrent use of clean and solid fuel stoves is pervasive in China (21, 39–42) and in many other countries where clean fuel use is growing (19, 27, 43–51). Clean fuels are increasingly accessible to and used by rural homes. Tracking exclusive or near-exclusive clean fuel use may be a more relevant household energy indicator, marking a major shift from recent decades. National-level surveys assess primary household fuel use, with a limited number of surveys collecting information on secondary fuels. Country-level census or surveys (e.g., Living Standard Measurement Surveys and Demographic and Health Surveys) could include questions on the retirement of solid fuel stoves for specific activities (i.e., cooking, heating, lighting). This information would allow for more accurate estimation of the health burden associated with household energy and better tracking of progress toward SDGs. This information would also shed new light on disparities in exclusive use of clean fuel, alongside indicators of access to clean fuels and technologies. For example, the World Health Organization in partnership with the United Nations Energy and Sustainable Energy for All (SE4ALL) Global Tracking Framework tracks the proportion of the population *primarily* using clean fuels as part of documenting progress on SDG 7. Follow-on questions on the frequency of solid fuel use, like we see emerging in the Multi-Tier Tracking Framework (also for tracking SDG 7 progress) from the Energy Sector Management Assistance Program (ESMAP), a program within the World Bank’s Energy and Extractives “Global Practice”, would improve country-level tracking of household energy transition.

Our study evaluated a comprehensive set of household-level factors (i.e., household size and composition, socioeconomic status, and cooking and heating behaviors) and a number of distal factors (i.e., geography and urbanicity) that have been previously associated with the adoption and use of clean fuels (29, 31, 42). Compared with the lowest income group (n=101; <2,500 RMB per year), higher income at all levels was associated with the greater uptake of clean cooking fuel in our study. However, income level was not associated with suspension of solid cooking fuel, with the exception of the very highest income bracket (n= 250; >35,000 RMB per year). This finding may reflect the choice by higher-income households to increase cooking intensity, rather than cease solid fuel use, when adopting clean fuels (15, 52). Households with the very highest income may be uniquely capable of achieving their desired cooking energy intensity using exclusively clean fuels. We found some evidence that clean energy transitions may be more likely to occur with other major life transitions, including work retirement or death of a spouse. These changes may result in smaller households, which was also associated with clean fuel adoption. Younger age was associated with both uptake of clean cooking fuel and suspension of solid cooking fuel and could reflect a greater willingness to discontinue traditional cooking practices.

It is perhaps not surprising that the set of factors associated with clean fuel uptake in our study were different than solid fuel suspension. Achieving complete transition to clean fuels requires households to not only adopt clean stove technologies but to also ‘give up’ the solid fuel stoves that they have used throughout their lifetimes. Borrowing from the sociotechnical frameworks developed to accelerate low-carbon transitions, for example, the availability of innovative technologies (i.e., clean fuels and stoves) is crucial, but complete transition to clean household energy requires a weakening of the existing systems that support solid fuel use (i.e., ‘phase out’ policies like targeted financial incentives) and the existence of strong exogenous pressures (i.e., development of new social preferences) to which households and communities feel compelled to respond (53, 54). Future studies on this topic would benefit from comparative studies, and particularly multi-country studies, where sites vary by these factors.

At a provincial level, a higher prevalence of clean fuel uptake did not correspond to higher rates of solid fuel suspension in our study. This discrepancy may be partially explained by community-scale energy transitions, which were more pronounced for suspension of solid fuel than for uptake of clean fuel (Supplementary Figures 3 and 4). For example, in Shanxi, 68% of households suspending use of solid cooking fuels were from 2 villages. In 1 of these villages, 92% and 87% of study participants who suspended use of solid fuel for cooking and heating, respectively, did so somewhat recently (within the past 5 years). The higher suspension in this village was likely attributable to a concurrent housing redevelopment that fully integrated piped natural gas into all homes for all villagers. This finding supports a broader literature on socio-technological transition showing that sustained change requires investment in new infrastructures, establishment of new markets, and adjustment of user practices (55). For example, India recently expanded LPG coverage to over 50 million low-income households through an innovative policy that targeted LPG subsidies more precisely to poor households and away from middle- and higher-income consumers (56). Clean energy transitions can also be accelerated by actively phasing out existing technologies, supply chains, and other systems that ‘lock in’ use of polluting technologies (57). In the UK, for example, household transition to gas was accelerated by the 1956 Clean Air Act, which restricted coal use in people’s homes and enabled cities to create smokeless areas that banned coal use entirely (58). More recently, destruction of traditional stoves or bans on household coal use have been implemented alongside new stove technologies to reduce household and outdoor air pollution in India and China (59–61), two countries where clean fuels are increasingly accessible (13, 30).

Unique strengths of our study include our ability to leverage a multi-provincial cohort of Chinese adults, which increases the generalizability of our findings within China and potentially to other regions of the world where clean fuel use is increasing but solid fuel use persists. Our use of an image-based questionnaire allowed us to comprehensively assess the diversity of household fuels and energy appliances used, their purpose, and levels of use over time. This tool was straightforward to develop, adaptable to different settings, relatively quick to implement with adult participants of all ages, and successfully captured information on suspension of solid fuels. We also find that the framework for our questionnaire reflects that of the Multi-Tier Tracking Framework for household energy use, which has emerged since the time of the present study. This tool was developed by the Energy Sector Management Assistance Program, in partnership with the World Bank, and reflects the need for more comprehensive household energy use tracking, as increasing evidence shows the energy ladder inadequately reflects recent real-world practices. With further testing and validation in a wider range of settings, locally-contextualized, image-based questionnaires, such as the one developed for this study, could potentially support future wider-spread household energy tracking efforts.

Our study was also subject to several limitations to consider in future studies. Self-reported stove and fuel use since baseline were retrospectively collected and thus subject to recall bias. To address this, we collected information in a standardized way with all participants. In villages where government officials had records of infrastructure change (i.e., timing of installation of new gas lines), we verified that the participant-reported information matched these records and also verified survey results through home assessments in a subset of homes. Successfully cross-referencing participant responses when additional village-level information was available provided evidence that study participants were capable of reliable recall of their household energy use history as captured by our questionnaire. Our analysis is also subject to omitted variable bias by factors that we were unable to measure but may influence household energy transitions, including household knowledge and perceptions, changes in income between baseline and follow up, fuel technology performance (e.g. efficiency), as well as changes over time in fuel supply, cost, and local energy policy and management. Village of residence was an important determinant in our models, indicating that extensions of this work could investigate the role of these factors.

## Conclusions

In this first study of both clean fuel adoption and solid fuel suspension, we found that use of clean stove technologies has dramatically increased over the past two decades in our cohort of 753 rural Chinese households. Subsequent transition to exclusive clean fuel use was comparably less common and slower, even among the households using clean fuels for decades. We also found that the set of village and household-level factors associated with solid fuel suspension and its timing differed from clean fuel uptake, a result that can help inform the planning, prediction, and evaluation of sustainable energy transitions in China and other low-income countries. Our study extends the existing clean energy transition literature by evaluating the factors that contribute to suspension of solid fuel stoves, which is an essential component of the clean energy transition process. Given the emerging value placed on displacement of solid fuels in poor and rural communities (11, 62), we show that solid fuel suspension warrants further study in diverse settings to reduce uncertainties when setting national, regional, and local energy policy priorities and allocating resources.

## Methods

The study design and participants of the ICP study are described elsewhere (63, 64). Briefly, 839 adults (50% female, ages 40-59) from rural areas of Beijing, Shanxi, and Guangxi were randomly selected and enrolled into the cross-sectional International Study of Macro/Micronutrients and Blood Pressure (INTERMAP) between 1995-1997. These sites (Supplementary Figure 5) were chosen to represent low-income areas and rural populations that were characteristic of northern and southern China. At baseline, all participants used solid fuel stoves for cooking or heating (or both). From 2015 to 2016, we re-enrolled 574 (85%) of the 680 surviving INTERMAP participants into the ICP study (Supplementary Table 1). In 2015-2016, an additional 210 individuals ages 40-59 were randomly selected from the same study villages (i.e., the study villages from the original INTERMAP study) and recruited into the study (Supplementary Table 1) to evaluate cohort differences in environmental and nutritional risk factors over time. Ethical approvals were obtained from review boards in China, the United Kingdom, and Canada. All subjects provided informed consent to participate in this study. To note, two of the individuals who enrolled and completed the household energy use questionnaire did not ultimately complete sufficient measurements to be included in health, exposure, and other sub-studies associated with the overall ICP study. Thus, the total sample size enrolled in this study was 784, while the overall ICP study size is reported as 782 (cite).

### Data collection

Structured questionnaires were administered by trained field staff at baseline and follow-up visits to collect information on age, education, ethnicity, occupation, marital status, household membership, socioeconomic status, and fuel and energy use practices (ICP study only). In-person interviews were conducted at centrally located clinics in each village. To reduce loss to follow-up, 9 participants from Beijing and 7 participants from Shanxi were interviewed by phone in July 2018. Detailed descriptions of the measurements conducted during the INTERMAP and ICP studies are published elsewhere (63, 65).

### Measures of current and historical household energy use

We administered an image-based questionnaire to collect information on the uptake, use, and suspension of all types of household energy devices and fuels since baseline assessment (Supplementary Figure 1). Detailed information on questionnaire development is provided in the SI text. For each device pictured, participants indicated whether they had used it in the past 20 years and, if so, they responded to the following questions: 1) “When did you start using the device?”; 2) “When did you stop using the device?”; 3) “Where in the home is/was the device used?”; 4) “With what frequency is/was the device used?”; 5) “For what purpose is/was the device used?”; and 6) “With what fuel(s) is/was the device used?”. Possible responses are provided in Supplementary Table 4.

Devices were subsequently classified into one of the following categories: solid fuel cooking stoves, gas cooking stoves, electric cooking appliances, solid fuel heating stoves, *kang* (bed) heating stoves, and electric heating appliances. Water heating devices were classified into cooking categories or heating categories based on participant activity responses (e.g., boiling water on a stove that is used to heat the room was classified under heating; heating water to wash cooking pots was classified under cooking). From these stove and fuel categories, and the corresponding usage patterns participants reported, we then constructed a set of categorical variables (Supplementary Table 5) to characterize and model energy use patterns over time.

While we did not encounter any responses that reflected a lapse in any fuel use for cooking or heating, the structure of our questionnaire would have allowed for such responses. In rare instances that a household started using a technology, stopped, and then re-started, our questionnaire was structured to capture these changes. At least in this study, changes in use of individual devices did not impact our results because it did not change the overall fuel composition status for the participant, likely due to energy stacking.

We also did not encounter an instance where a participant response flagged for verification proved to be reported in error, even for some responses that were particularly unusual or uncommon. For example, in Beijing, a small number of participants (n~4) reported having centralized, electric, radiant floor heating. This was a new response we had not previously encountered for space heating and unusual. We followed up by visiting these homes, which allowed us to verify that these homes were uniquely set up with radiant floor heating.

### Statistical analysis

We evaluated the household- and community-level factors that influence the household uptake of a clean fuel, the suspension of solid fuel, and the timing of those decisions using Cragg’s double hurdle models (66). These models separate the household energy transition process into two parts, namely the decision to suspend use of solid fuel or adopt clean fuel and the timing at which the household made those decisions. They were specifically developed to analyze censored dependent variables (i.e., households that start using clean fuels or stop using solid fuel before the end of the study) (67), whereas an ordinary least squares regression model would yield biased estimates.

In practical terms, in the first stage of the double hurdle model, the problem is to estimate P(*Q* = 1 | θ), the probability that *Q* = 1 (that a household suspended use of solid fuel or started using clean fuel) conditional on an observed set of covariates θ. Taking this into account, we then estimated E(*t* | θ, *t* > 0) using a truncated regression model. An assumption of this two-part model is that *Q* and $t_{ij}^{*}$ are independent, conditional on explanatory variables θ, but we can include all variables θ in both the first and second-stage equations while allowing the parameters on those variables to freely vary between equations (67). In this case, we considered θ to contain the household and community factors listed above and employed a probit specification to model the probability of solid fuel suspension and clean energy uptake as a function of these variables. The probit regression coefficients are not as directly interpretable as those from a linear or logistic regression model. The increase in probability attributed to a one-unit increase in an independent variable in a probit regression is dependent on the values of all other independent variables and their intial conditions. We can interpret a positive coefficient to indicate that an increase in the variable, or a state other than the reference state, is associated with an increase in the predicted probability of the outcome. Conversely, a negative coefficient would indicate that a decrease in the variable, or a state other than the reference state, is associated with a decrease in the predicted probability of the outcome.

We modeled 4 dependent variables: 1) time since most recent suspension of solid cooking fuel, 2) time since most recent suspension of solid heating fuel, 3) time since earliest uptake of clean cooking fuel, and 4) time since earliest uptake of clean heating fuel. Independent variables were selected *a priori* based on the stove adoption and energy transition literature (31, 42), and included the following in 2016: age (integer); household size (integer); marital status (married, widowed); educational attainment (no school; primary school; and early high school / college); occupation (retired, agricultural work, non-agricultural work); income (units of *renminbi* (RMB): <2,500; 2,500-4,999; 5,000-9,999; 10,000-19,999; 20,000-34,999; >35,000), and self-reported health status (excellent, good, fair, poor). For INTERMAP participants that enrolled into the ICP study (n=575), some independent variables (i.e., age, marital status, educational attainment) were collected at baseline (1997) and follow-up (2016), while others were collected at follow-up only (i.e., income, household size, occupation, self-reported health). For educational attainment there was no change over the 20-year follow-up period. For marital status, approximately 14% of participants enrolled at baseline and follow up (n=79) experienced a change, which for more than 83% of those participants was to become widowed. In both cases, this is likely due to the age at which we enrolled participants at baseline (40-59 years), which was well into adulthood. Among newly-enrolled ICP study participants who did not participate in INTERMAP (n=209), all of the independent variables represented in our models were collected in 2016. To control for factors that were constant within the two age cohorts (e.g., INTERMAP participants enrolled in 1997-1998 versus ICP participants newly enrolled in 2015-2016) we included a dummy variable for enrollment status. To control for factors that were constant within individual communities (e.g. infrastructure, village average purchasing power, prices, and other unobserved community attributes jointly correlated with the included regressors and outcomes), we included factor variables to represent the village of residence. Approximately 10% of participants reported that they either did not know or did not wish to disclose their income, so we imputed their income level (at follow-up, i.e., 2016) based on all other available data using R packages for visualization and imputation of missing data (vim) and multivariate imputation using chained equations (mice) (cran.r-project.org).

For each dependent variable, we considered a binary variable *Q* that determines whether household *i* ceases to use solid fuel (or starts using clean energy) in year *t* or never. When choosing when to cease (or begin) fuel use, household *i* in village *j* appears to follow $t_{ij}=Q\cdot t_{ij}^{*}$, where $t_{ij}^{*}$ is a continuous latent variable. Thus, the observed time to cessation variable, *t*, is a limited dependent variable that is censored at 0 years for households still using solid fuels (or never taking up clean energy). Households that suspended use of solid fuels (or started using clean energy) take on “true” values of 5, 10, 15, or 20 years since transitioning in the past. When a household transitions (*Q* = 1), $t_{ij}=t_{ij}^{*}$.

As sensitivity analyses, we re-analyzed the data using the same models, but with households reporting ‘rare use of solid fuel’ being classified as solid fuel users, whereas they were classified as clean fuel users in the main analysis. We also repeated our analysis pooling the cooking and heating outcomes. Thus, we modeled suspension and uptake of any solid or clean fuel and time since most recent suspension or uptake of any solid or clean fuel, regardless of activity.

Statistical analyses were performed in Stata 13 (StataCorp LP) and R (cran.r-project.org).

## Supplementary Material

Supplementary Material

## Figures and Tables

**Table 1:** Proportion (and number) of participants reporting suspension of solid fuel and uptake of clean fuel over the 20-year reporting period.

Household energy variables	All sites (n=753)	Beijing (n=246)	Shanxi (n=284)	Guangxi (n=223)
Uptake of clean energy				
Cooking	95% (717)	98% (241)	91% (259)	97% (217)
Heating	73%* (389)	90% (221)	59%(168)	6% (14)
Suspension of solid fuel				
Cooking	35% (266)	30% (73)	44% (125)	30% (68)
Heating	17%* (90)	9% (21)	20% (55)	NA

* Denominator includes only participants in northern China (n=530)

**Table 2:** Determinants of **whether** households suspended use of solid fuels or started use of clean energy. The table reports the modelled, first-stage coefficients for each variable (zero-hurdle model: binomial with probit link).

Variables	Solid Fuel Suspension		Clean Fuel Uptake	
	Cooking	Heating	Cooking	Heating
Age	-0.02 (0.01)*	-0.004 (0.01)	-0.06 (0.02)**	-0.004 (0.01)
Number of people in household	-0.03 (0.04)	-0.06 (0.05)	-0.17 (0.06)**	-0.05 (0.04)
Time since uptake of clean cooking fuel	0.06 (0.01)**	-0.04 (0.02)*	NA	NA
Time since uptake of clean heating fuel	-0.03 (0.01)*	0.06 (0.01)**	NA	NA
Cohort (ref: original participants)	0.27 (0.26)	0.06 (0.24)	0.14 (0.50)	-0.20 (0.25)
Income (ref: <2,500 RMB^a^)				
2,500 - 4,999	0.27 (0.29)	-0.03 (0.33)	1.13 (0.63)*	-0.23 (0.28)
5,000 - 9,999	0.42 (0.26)	-0.39 (0.30)	0.93 (0.44)*	-0.37 (0.23)
10,000 - 19,999	0.06 (0.25)	0.29 (0.26)	1.29 (0.45)*	-0.04 (0.23)
20,000 - 34,999	0.39 (0.23)	-0.08 (0.25)	0.89 (0.39)*	-0.12 (0.22)
>35,000	0.62 (0.24)**	-0.26 (0.31)	0.57 (0.40)	0.49 (0.30)
Marital status (ref: widowed)				
Married	-0.37 (0.16)*	-0.05 (0.25)	-0.29 (0.30)	0.03 (0.22)
Education (ref: no school)				
Primary school	-0.01 (0.16)	0.14 (0.26)	-0.15 (0.29)	0.03 (0.19)
Early high school / college	-0.05 (0.18)	0.45 (0.26)	0.43 (0.38)	0.44 (0.22)*
Occupation (ref: retired)				
Agricultural work	-0.12 (0.15)	-0.34 (0.19)	-0.30 (0.33)	-0.02 (0.18)
Non-agricultural work	0.11 (0.23)	0.07 (0.33)	-1.07 (0.48)*	-0.63 (0.32)*
Self-reported health status (ref: excellent)				
Good	-0.15 (0.21)	0.51 (0.30)	-0.11 (0.43)	-0.31 (0.25)
Fair	0.17 (0.20)	0.46 (0.29)	0.08 (0.43)	-0.31 (0.25)
Poor	0.11 (0.22)	0.15 (0.35)	-0.37 (0.46)	-0.11 (0.30)

* indicates a p-value ≤0.05

** indicates a p-value ≤0.01

a RMB is *renminbi*, Chinese currency (~0.15 USD)

Note: While an increase in the probability of the outcome attributable to a one-unit increase in a given independent variable in the probit regression is dependent on the values of all other independent variables and their initial conditions, we can interpret a positive (negative) coefficient to indicate that an increase (decrease) in the variable, or a state other than the reference state, is associated with an increase (decrease) in the predicted probability of the outcome.

**Table 3:** Determinants of **when** households suspended use of solid fuels or started use of clean energy. The table reports the modelled, second-stage coefficients for each variable (count model: truncated Poisson with log link).

Variables	Solid Fuel Suspension		Clean fuel Uptake	
	Cooking	Heating	Cooking	Heating
Age	-0.01 (0.004)**	-0.01 (0.001)	-0.003 (0.001)	-0.002 (0.003)
Number of people in household	-0.01 (0.02)	-0.00 (0.004)	-0.01 (0.01)	-0.02 (0.01)
Time since uptake of clean cooking fuel	0.03 (0.01)**	-0.03 (0.001)**	NA	NA
Time since uptake of clean heating fuel	-0.001 (0.004)	0.05 (0.01)**	NA	NA
Cohort (ref: original participants)	0.04 (0.10)	0.04 (0.01)*	-0.09 (0.05)*	-0.12 (0.07)
Income (ref: <2,500 RMB^a^)				
2,500 - 4,999	0.003 (0.12)	0.04 (0.02)	0.01 (0.05)	-0.15 (0.07)*
5,000 - 9,999	-0.05 (0.10)	-0.003 (0.02)	-0.02 (0.05)	0.002 (0.06)
10,000 - 19,999	-0.10 (0.10)	0.01 (0.02)	0.01 (0.04)	-0.18 (0.06)*
20,000 - 34,999	-0.09 (0.09)	-0.002 (0.02)	0.02 (0.04)	-0.18 (0.06)*
>35,000	-0.07 (0.10)	0.01 (0.02)	0.09 (0.04)*	-0.09 (0.06)
Marital status (ref: widowed)				
Married	0.08 (0.08)	0.01 (0.02)	0.05 (0.03)	-0.02 (0.06)
Education (ref: no school)				
Primary school	0.14 (0.08)	-0.01 (0.02)	-0.002 (0.03)	-0.02 (0.05)
Early high school / college	0.17 (0.09)*	0.02 (0.02)	0.02 (0.03)	0.05 (0.05)
Occupation (ref: retired)				
Agricultural work	-0.07 (0.07)	-0.02 (0.01)	-0.006 (0.03)	-0.04 (0.04)
Non-agricultural work	-0.13 (0.10)	-0.0048 (0.02)	-0.01 (0.04)	0.03 (0.07)
Self-reported health status (ref: excellent)				
Good	-0.04 (0.09)	0.06 (0.02)**	0.04 (0.04)	0.001 (0.05)
Fair	0.01 (0.09)	0.05 (0.02)*	0.01 (0.04)	0.07 (0.05)
Poor	0.04 (0.10)	0.04 (0.03)	-0.04 (0.04)	-0.10 (0.06)

* indicates a p-value ≤0.05

** indicates a p-value ≤0.01

a RMB is *renminbi*, Chinese currency (~0.15 USD)

## Data Availability

The data that support the findings of this study are available from the corresponding author upon request. Requests for datasets generated and analyzed during the current study will be reviewed and made available on a case-by-case basis by the corresponding author with input from co-authors, subject to compliance with Research Ethics Board restrictions for the survey data. Figures 1-4 and S1-S4 contain primary data.

## Code Availability

Requests for code developed and annotated in Stata 13 and R to process and analyze the primary data collected in this study will be reviewed and made available upon reasonable request.
