# Supplementary material for: Household Transitions to Clean Energy in a Multi-Provincial Cohort Study in China
Source: Nat Sustain. Author manuscript; Available in PMC 2023 Sep 27. (PMC7615133; doi:10.1038/s41893-019-0432-x)
Supplement: Supplementary Material [file EMS188277-supplement-Supplementary_Material.pdf]

nature sustainability

SUPPLEMENTARY INFORMATION

https://doi.org/10.1038/s41893-019-0432-x

In the format provided by the authors and unedited.

Household transitions to clean energy in a multiprovincial cohort study in China

Ellison Carter[1,2*], Li Yan[3,4], Yu Fu[5], Brian Robinson[6], Frank Kelly[4], Paul Elliott[3,7], Yangfeng Wu[8], Liancheng Zhao[9], Majid Ezzati[3,7], Xudong Yang[5], Queenie Chan[3] and Jill Baumgartner[10*]

[1]Department of Civil and Environmental Engineering, Colorado State University, Fort Collins, CO, USA. [2]Institute on the Environment, University of Minnesota, Saint Paul, MN, USA. [3]Department of Epidemiology and Biostatistics, School of Public Health, Imperial College London, London, UK. [4]Department of Analytical, Environmental and Forensic Sciences, School of Population Health and Environmental Sciences, Kings College London, London, UK. [5]Department of Building Science, School of Architecture, Tsinghua University, Beijing, China. [6]Department of Geography, McGill University, Montreal, Québec, Canada. [7]MRC-PHE Centre for Environment and Health, School of Public Health, Imperial College London, London, UK. [8]Peking University Clinical Research Institute, Beijing, China. [9]National Center for Cardiovascular Disease, Fuwai Hospital, Peking Union Medical College and Chinese Academy of Medical Sciences, Beijing, China. [10]Department of Epidemiology, Biostatistics, and Occupational Health, McGill University, Montreal, Québec, Canada.
*e-mail: ellison.carter@colostate.edu; jill.baumgartner@mcgill.ca

Supplementary Material for

Title: Household Transitions to Clean Energy in a Multi-Provincial Cohort Study in China

This file includes:

Supplementary Figs. 1 to 5
Supplementary Tables 1 to 5

Other supplementary materials for this manuscript include the following:

None

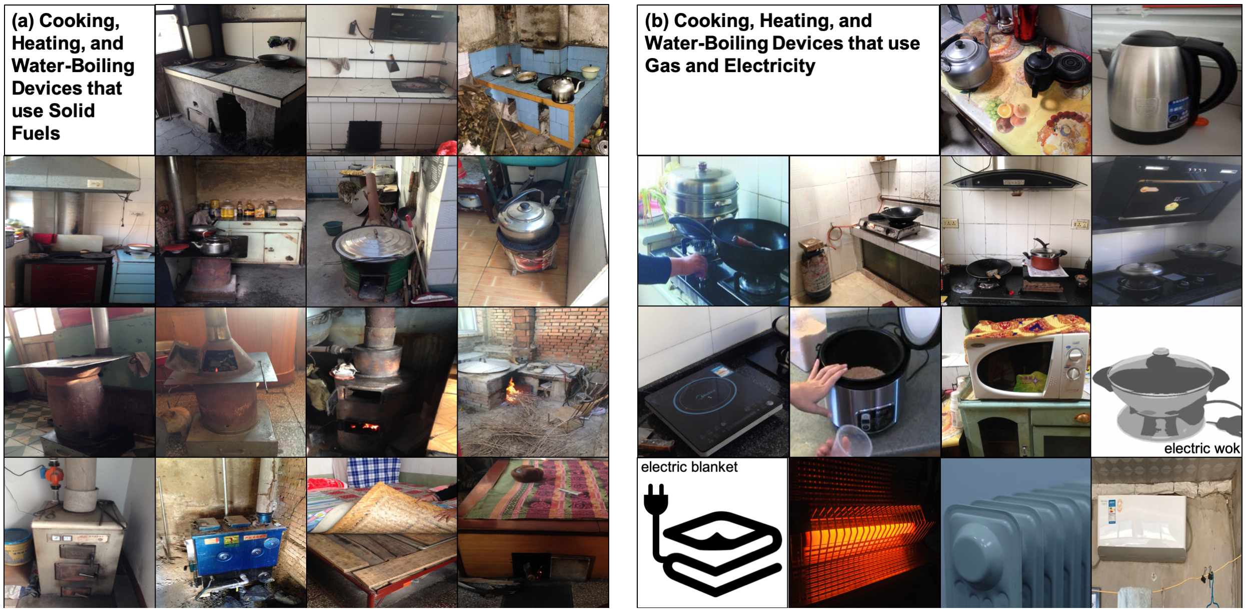

Supplementary Figure 1. Representative images of 15 distinct types of household cooking (n=7) and heating (n=8) devices that use solid fuel (a) and 14 distinct types of cooking (n=8), heating (n=4), and water-boiling (n=2) appliances that use gas or electricity (b) reported by participants.

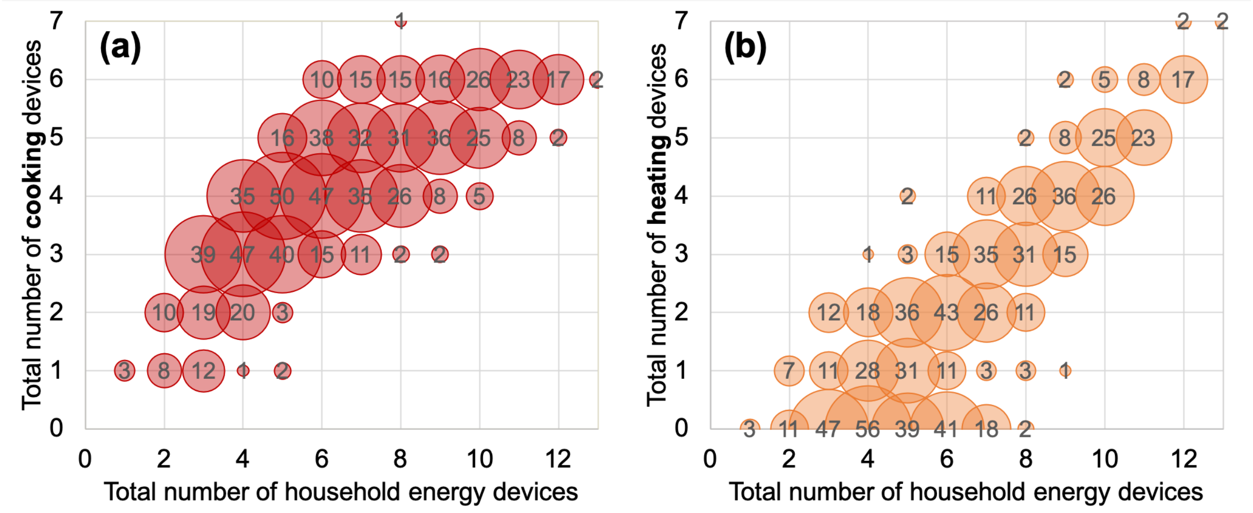

Supplementary Figure 2. Total number of cooking (a) or heating (b) devices owned as a function of total household energy devices reported. Marker size is proportional to the number of participants (indicated in the circle).

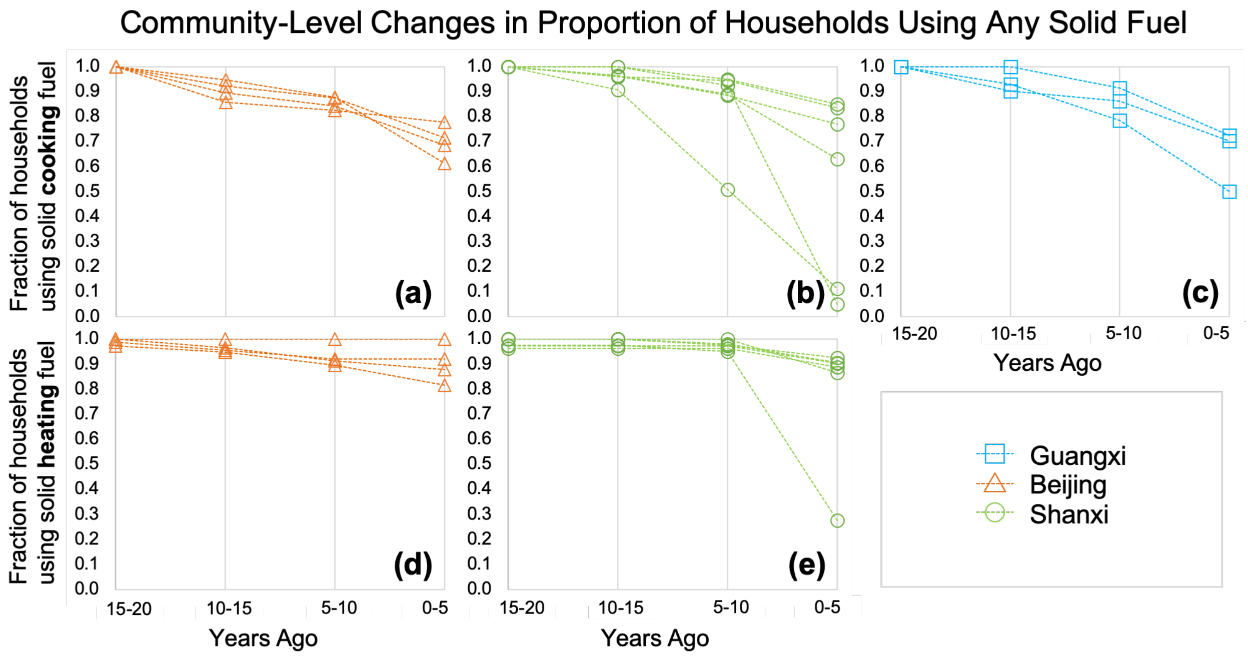

Supplementary Figure 3. Temporal trends, evaluated at 5-year intervals, in suspension of solid fuel for cooking activities shown separately for each village in Beijing (a), Shanxi (b), and Guangxi (c) and suspension of solid fuel for heating activities in Beijing (d) and Shanxi (e).

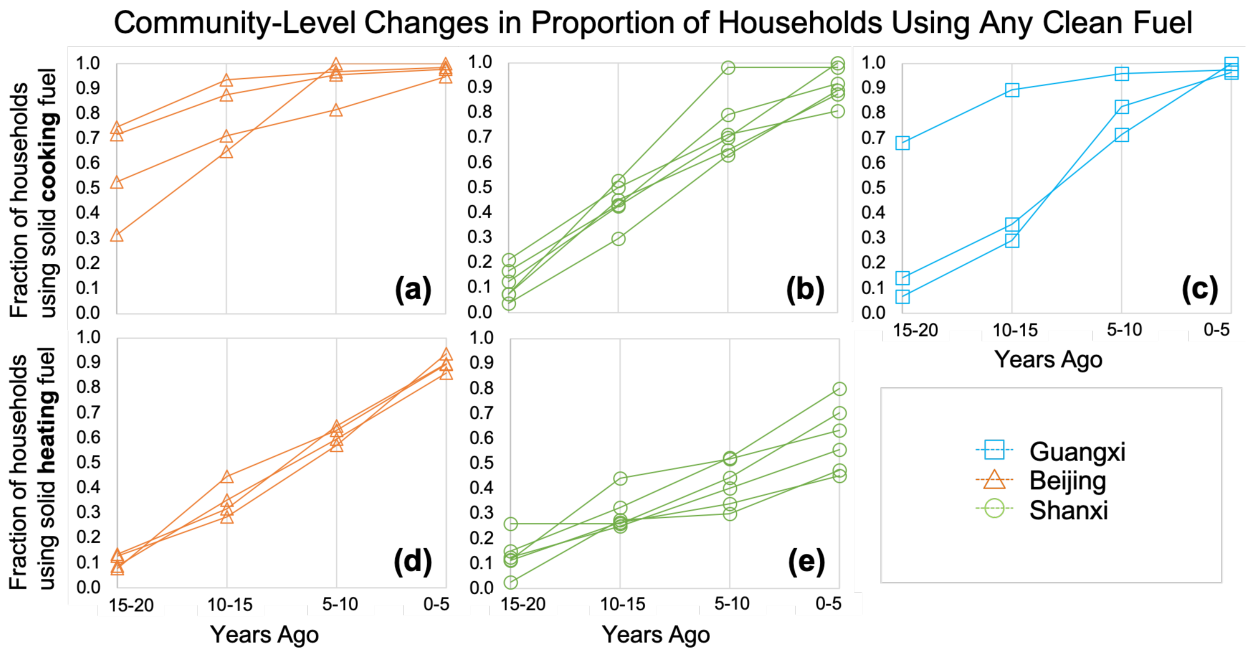

Supplementary Figure 4. Temporal trends, evaluated at 5-year intervals, in uptake of clean fuel for cooking activities shown separately for each village in Beijing (a), Shanxi (b), and Guangxi (c) and uptake of clean fuel for heating activities in Beijing (d) and Shanxi (e).

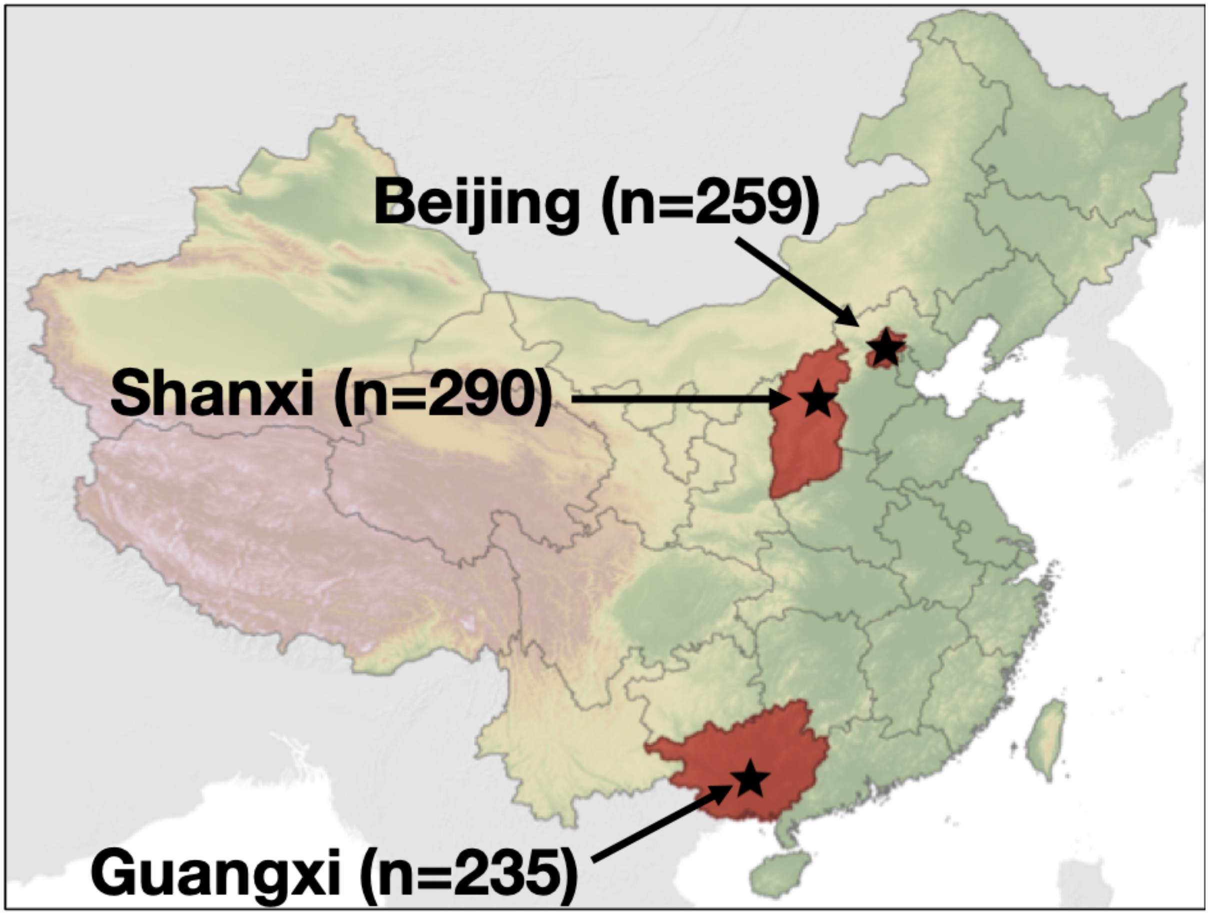

Supplementary Figure 5. Map of geopolitical provincial boundaries in China with field sites indicated for the 3 study provinces – Beijing, Shanxi, and Guangxi – where participants (n) were recruited into the present study.

Supplementary Table 1. Socio-demographic and household energy characteristics of ICP study participants [mean ± SD and range or number (%)].

Variables	Beijing (n=246)[a]	Shanxi (n=284)	Guangxi (n=223)
Age [mean ± SD (range)]	63.4 ± 7.6 (43-79)	61.9 ± 8.7 (40-78)	63.2 ± 9.5 (41-78)
Household membership[a]	2.8 ± 1.4 (1-7)	3.4 ± 1.9 (1-10)	3.1 ± 1.5 (1-7)
Ethnicity			
Han	244 (99%)	284 (100%)	114 (51%)
Other	2 (1%)	0 (0%)	109 (49%)
Marital status			
Married (past/present)	222 (90%)	251 (88%)	169 (76%)
Widowed[b]	24 (10%)	33 (12%)	54 (24%)
Educational attainment			
No school	59 (24%)	29 (10%)	26 (12%)
Primary school	79 (32%)	136 (48%)	97 (43%)
Early high school / college	108 (44%)	119 (42%)	100 (45%)
Occupation			
Retired	36 (15%)	55 (19%)	162 (73%)
Agricultural work	197 (80%)	209 (74%)	32 (14%)
Non-agricultural work	13 (5%)	20 (7%)	29 (13%)
Income (RMB[c])			
<2,500	5 (2%)	85 (30%)	9 (4%)
2,500 - 4,999	22 (9%)	22 (8%)	3 (1%)
5,000 - 9,999	44 (18%)	34 (12%)	2 (<1%)
10,000 - 19,999	55 (22%)	46 (16%)	13 (6%)
20,000 - 34,999	51 (21%)	71 (25%)	40 (18%)
>35,000	69 (28%)	26 (9%)	156 (70%)
Self-reported health status			
Excellent	29 (12%)	28 (10%)	10 (4%)
Good	77 (31%)	93 (33%)	56 (25%)
Fair	100 (41%)	132 (46%)	108 (48%)
Poor	40 (16%)	31 (11%)	49 (22%)
Household energy devices [mean ± SD (range)]			
Total	8.7 ± 2.2 (2-13)	5.8 ± 1.8 (2-11)	4.6 ± 1.5 (1-9)
Cooking devices	4.8 ± 1.2 (1-6)	3.6 ± 1.2 (1-6)	4.0 ± 1.3 (1-7)
Heating devices	3.9 ± 1.5 (0-7)	2.2 ± 1.1 (0-6)	NA[d]
Number of respondents (administrative units)	246 (8)	284 (6)	223 (3)

[a] two of the individuals who enrolled and completed the household energy use questionnaire did not ultimately complete sufficient measurements to be included in health, exposure, and other sub-studies associated with the overall ICP study.

Supplementary Table 2. Determinants of *whether* households suspended use of solid fuels and started use of clean energy. The table reports the modelled, first-stage coefficients for each variable (zero hurdle model: binomial with probit link). Cooking and heating outcomes are pooled. (*Please see Table S2 footnotes below for explanation of how to interpret first-stage model coefficients*.)

Variables	Solid Fuel Suspension	Clean Fuel Uptake
Age	-0.03 (0.01)**	-0.04 (0.02)
Number of people in household	-0.04 (0.04)	-0.05 (0.08)
Time since uptake of clean cooking fuel	0.03 (0.01)**	NA
Time since uptake of clean heating fuel	0.01 (0.01)	NA
Cohort (ref: original participants)	0.08 (0.27)	-0.34 (0.63)
Income (ref: <2,500 RMB[a])		
2,500 - 4,999 RMB	0.05 (0.29)	4.9 (801)
5,000 - 9,999 RMB	0.24 (0.25)	0.74 (0.57)
10,000 - 19,999 RMB	0.18 (0.24)	0.65 (0.53)
20,000 - 34,999 RMB	0.27 (0.23)	0.47 (0.45)
>35,000 RMB	0.52 (0.24)*	0.11 (0.51)
Marital status (ref: widowed)		
Married	-0.30 (0.15)*	-0.42 (0.37)
Education (ref: no school)		
Primary school	-0.03 (0.16)	-0.03 (0.34)
Early high school / college	0.01 (0.18)	0.79 (0.50)
Occupation (ref: retired)		
Agricultural work	-0.15 (0.15)	-0.17 (0.39)
Non-agricultural work	0.11 (0.23)	-1.12 (0.52)*
Self-reported health status (ref: excellent)		
Good	0.00 (0.21)	-0.17 (0.54)
Fair	0.23 (0.20)	-0.21 (0.53)
Poor	0.17 (0.22)	-0.37 (0.57)

Note: While an increase in the probability of the outcome attributable to a one-unit increase in a given independent variable in the probit regression is dependent on the values of all independent variables and their initial conditions, we can interpret a positive (negative) coefficient to indicate that an increase (decrease) in the variable, or a state other than the reference state, is associated with an increase (decrease) in the predicted probability of the outcome.

Supplementary Table 3. Determinants of *when* households suspended use of solid fuels and started use of clean energy. The table reports the modelled, second-stage coefficients for each variable (count model: truncated Poisson with log link). Cooking and heating outcomes are pooled.

Variables	Solid Fuel Suspension	Clean Fuel Uptake
Age	-0.01 (0.003)**	-0.002 (0.001)
Number of people in household	-0.01 (0.02)	-0.01 (0.007)*
Time since uptake of clean cooking fuel	0.03 (0.005)**	
Time since uptake of clean heating fuel	0.006 (0.003)	
Cohort (ref: original participants)	0.06 (0.09)	-0.12 (0.04)**
Income (ref: <2,500 RMB[a])		
2,500 - 4,999 RMB	-0.01 (0.11)	-0.04 (0.05)
5,000 - 9,999 RMB	-0.16 (0.09)	-0.02 (0.04)
10,000 - 19,999 RMB	-0.16 (0.09)	-0.01 (0.04)
20,000 - 34,999 RMB	-0.21 (0.08)**	-0.02 (0.04)
>35,000 RMB	-0.18 (0.09)*	0.05 (0.04)
Marital status (ref: widowed)		
Married	0.10 (0.07)	0.04 (0.03)
Education (ref: no school)		
Primary school	0.12 (0.07)	0.01 (0.03)
Early high school / college	0.19 (0.08)*	0.04 (0.03)
Occupation (ref: retired)		
Agricultural work	-0.10 (0.06)	-0.01 (0.03)
Non-agricultural work	-0.14 (0.09)	-0.02 (0.04)
Self-reported health status (ref: excellent)		
Good	0.03 (0.08)	-0.02 (0.04)
Fair	0.05 (0.09)	-0.02 (0.04)
Poor	0.04 (0.09)	-0.08 (0.04)

Supplementary Table 4. Questions and responses in the household stove and fuel questionnaire.[a]

Question	Responses				
Do you now or have you ever used this device?	Yes	No			
When did you start using this device? (years ago)	0-5	5-10	10-15	15-20	
When did you stop using this device? (years ago)	Have not stopped	0-5	5-10	10-15	15-20
What do you or did you use this device for?	Cooking	Heating	Cooking and heating	Water heating and space heating	
Where do you or did you use this device?	Living room	Bedroom	Kitchen	Outside, attached, separate entrance	Outside, detached
How frequently do you or did you use this device?	Seldom<br>Holidays or when hosting many people<br>Only colder months<br>Only warmer months<br>2-5 hrs/day<br>14-16 hrs/day<br>24 hrs/day<br>Weekly<br>Daily				
What fuels do you or did you use with this device? (select all that apply)	Responses reflected local fuel options, including multiple types of coal, biomass, wood, crop residues, gas (LPG and biogas), and electricity.				

[a]To develop the questionnaire, we visited a representative sample of at least 10 homes in each study region and photographed all household energy devices and fuels present in the homes. We stopped visiting homes when no new devices or fuels could be identified and after verifying with local staff that our image database captured all known fuel-stove combinations used by homes in that region. A visual library of household stoves, energy appliances, and fuels was created for each site (**Supplementary Material Figure 1**). The questionnaire was iteratively field tested in homes prior to data collection in each province and administered by staff in the local dialect of Mandarin-Chinese. We engaged participants in a retrospective evaluation of their fuel use behaviour over a 20-year period. To minimize bias in responses and leverage as much information as possible (76), we asked participants to estimate times to the nearest 5-year window and select from a set of categorical responses.

Supplementary Table 5. Outcome measures developed to characterize historical and current energy use.

Variables	Description
Baseline energy use (1997)	Classified as one of the following:<br>1) Exclusive use of clean fuel<br>2) Mixed use of solid and clean fuel<br>3) Exclusive use of solid fuel<br>4) No use of solid or clean fuel
Current energy use (2016)	Classified as one of the following:<br>1) Exclusive use of clean fuel<br>2) Mixed use of solid and clean fuel<br>3) Exclusive use of solid fuel<br>4) No use of solid or clean fuel
Time since suspension of solid fuel	Time (in 5-year intervals) since the participant's most recent reported suspension of solid fuel. Determined separately and jointly for cooking and heating fuels.
Time since earliest uptake of clean fuel	Time (in 5-year intervals) since the participant's earliest reported uptake of clean fuel. Determined separately and jointly for cooking and heating fuels.
Frequency of use	Classified according to the following categories:<br>1) Rare* (seldom or for infrequent special occasions)<br>2) Seasonal (if used only in colder or warmer months)<br>3) Weekly (a few days/week or 3-4 times/week)<br>4) Daily (a number of hours per day or "all/every day")

*Switching to rare use of a solid fuel stove (n=29) was classified as suspending use for the main statistical analysis. As a sensitivity analysis, we re-ran the analysis classifying those participants as solid fuel users.
